# Applications of identity-based theories to understand the impact of stigma and camouflaging on mental health outcomes for autistic people

**DOI:** 10.3389/fpsyt.2023.1243657

**Published:** 2023-09-06

**Authors:** Rachel A. Rivera, Loisa Bennetto

**Affiliations:** Department of Psychology, University of Rochester, Rochester, NY, United States

**Keywords:** autistic identity, minority stress, social identity theory, camouflaging, stigma, mental health

## Abstract

Autistic people have long been conceptualized from a deficit-based model of disability, but recent self-advocates and scholars have asserted the importance of recognizing autism as both a disability and an important part of a person’s social identity. The autistic identity is subject to specific stigma and stressors beyond everyday discrimination and prejudice, which can have many downstream implications on mental health and well-being. Prior research on camouflaging has explained both quantitatively and qualitatively how autistic people conform to norms and mask their autistic traits to better fit in with non-autistic societal standards. Given this paradigm shift in understanding autistic peoples’ lived experiences, researchers must also begin to reshape the theories guiding their work in order to improve diagnosis, intervention, and supports. This review examines the extant research on identity-related stigma and camouflaging and their subsequent impacts on mental health outcomes in autism. A model is proposed integrating identity-based theories—specifically the social model of disability, social identity theory, and minority stress model—to explain relationships across research areas and better explain the experiences of autistic people. We discuss how identity-based theories can be applied in autism research to better understand the impacts of stigma and camouflaging on autistic peoples’ lived experiences and reduce disparities in their mental health outcomes.

## Introduction

Autistic people have various strengths and challenges in different domains such as language, social skills, executive functioning, sensory sensitivity, and focused interests and behaviors (1), and autistic traits can range in frequency and intensity across these areas (2). Since the turn of the century, advocacy efforts led by the autistic community have reshaped our understanding of autism, research priorities, and clinical practices (3) and have bolstered a sense of autistic identity and pride that confers a positive protective factor for self-esteem (4).

In addition to the strengths and challenges associated with autism, autistic people experience a multitude of mental health concerns at disproportionate rates to non-autistic people (5). While co-occurring psychopathology may result from within-person increased susceptibility [e.g., (6)], other external factors such as lack of accessibility to services (7), external stress (8), and discrimination based on disability status (9) can impact the likelihood of a person developing depression, anxiety, suicidality, or other mental health concerns. Stigmatization based on autistic identity can additionally contribute to stress and decreased mental health and well-being (10). Some autistic people may choose to hide their autistic traits and abide by social norms (known as camouflaging) to gain acceptance and avoid judgment from peers while others may experience prejudice and discrimination based on expressing their autistic characteristics and behaviors (11, 12).

It is crucial to understand how social dynamics influence how autistic people are perceived and treated by non-autistic people and subsequently, how autistic people act in response to this. Several social and identity-based theories have been proposed to explain how out-groups experience differential treatment or stress, including social identity theory (13) and the minority stress model (14); however, identity-based theories have not been commonly applied to research on understanding disabled peoples’ experiences. Elucidating this process can help ascertain how to best support autistic people and mitigate stress. Additionally, this can assist in highlighting how caregivers and professionals can target and change more systemic structures that contribute to prejudice and stigma. Moreover, topics like improving access to healthcare and mental health support, the impact of co-occurring mental health diagnoses, and the effects of stressful social environments and discriminatory systems have been identified as a chief priority for future research (15, 16).

## Aims

To our knowledge, there have only been two empirical studies examining the application of the minority stress model or social identity theory to understanding how stigma and camouflaging influence autistic peoples’ mental health (10, 17). Thus, the primary aim of the present integrative theoretical review was to synthesize the research literature from the following areas: stigma, camouflaging autistic traits, models of disability, social identity theory, and minority stress model. Moreover, this review had the secondary aim of incorporating these areas together to advocate for further research on the impacts of minority stress on autistic peoples’ camouflaging and mental health-related outcomes. In the sections below, we review stigma and camouflaging in autism and how we can use identity theories to better understand the subsequent difficulties and disparities and how to address them in structural systems and clinical practice.

### Autism-related stigma

While developing a positive sense of autistic identity can have benefits for self-esteem and community belongingness, autism is still stigmatized by society. Stigmatization is often a result from the process of labeling or disclosing one’s minority identity in a manner that negatively affects their mental health or emotional, physical, or social well-being. Autism stigma was originally conceptualized as the interplay between societally unacceptable social behavior with no noticeable physical markers of the disability and a general lack of public awareness about autism (18). As time progressed, additional research asserted that even with increased general autism knowledge, autism stigma evidently persisted (19, 20). This was posited to be primarily a result of autistic peoples’ behaviors within social interactions that result from difficulties and differences within their verbal and non-verbal social communication skillset (21–23) and secondarily a contribution of the stereotypes associated with an autism diagnostic label (24–26).

For autistic people, labeling their autism may lead to comparisons to their non-autistic peers and subsequent meaning-making of those differences (27, 28). While autistic people may not initially assign negative attributions to their identity, societal norms and opinions can assign negative values to the labels and consequently propagate stigma towards this group of people (29). For example, one study demonstrated that non-autistic individuals rated 9 out of 10 descriptions of autistic people as negative (30), and another study elucidated that many of the core autism traits and characteristics were stigmatized by participants (21). Consequences from stigma can be pervasive and detrimental to the well-being and self-worth of the marginalized and stigmatized group (31). Research has found that the autistic community experiences many difficulties in different areas of well-being including physical and sexual victimization across the lifespan (32, 33), workplace discrimination (34), and social rejection (31). A systematic review and meta-analysis from Lai and colleagues (5) also assessed the co-occurrent rates of mental health diagnoses within the autism population and reported high prevalence rates for diagnoses including attention-deficit hyperactivity disorder, anxiety disorders, depressive disorders, and disruptive, impulse-control, and conduct disorders, among others. Cage and colleagues (35) found that decreased external and personal autism acceptance significantly predicted depressive symptoms, and decreased acceptance from others predicted greater stress. Furthermore, several recent studies have also directly linked autism-related stigma to decreased mental health and well-being (10, 17, 36).

A recent literature review created the first theoretical model of autism stigma and what may contribute to it, moderate it, and result from it (37). Their model asserted that autism stigma is predominantly influenced by a combination of others’ interpretation of a person’s autistic traits and a lack of public and professional understanding of autism. Additional suggested moderators included identity-based factors like gender, sex, and cultural factors, diagnostic disclosure and individual differences, and finally, the frequency and quality of contact with autistic people. While there is a rich breadth of literature that emphasizes the high levels of mental health co-occurrence and poor well-being outcomes for autistic people, this research area remains relatively new and understudied (37). There is a strong need for researchers to collaborate with autistic self-advocates on how to better examine how stigma subsequently impacts autistic peoples’ thoughts and behaviors, identify the mechanisms that lead to autism stigma, and find creative solutions to decrease the perpetuation of autism stigma (38). An additional consideration is to conduct research on self-perceptions of peoples’ autistic identity. For some people, autism can be an invisible disability which is defined as any combination of physical, mental, or neurological differences that cannot be seen by others but still impacts day-to-day life (39). Additionally, autism can be an invisible identity which is a social identity that cannot be easily determined from visible cues (40). Therefore, for people whose autism is both an invisible disability and identity, subsequent research should investigate how people may actively change how they present themselves to avoid any anticipated or internalized stigma (41, 42).

### Camouflaging autistic traits

Individuals with more invisible stigmatized or minoritized identities typically choose one of two primary coping strategies: either disclose their identity or hide their identity from others (43, 44). Some may conceal their autistic identity by choosing not to disclose their diagnostic status, other people hide their autistic identity by abiding by social norms thus hiding (camouflaging) their autistic traits, and some may choose to utilize both strategies to avoid potential stigma [see (45) for a review]. Cage and Troxell-Whitman (46) found that having a stronger sense of autistic identity made autistic people less likely to engage in camouflaging behavior when they had greater disclosure. Camouflaging allows autistic people to minimize the differences between their behaviors and those of non-autistic people that they interact with on a daily basis (47–49). Some identified camouflaging strategies include suppressing self-stimulatory behaviors, mirroring non-autistic behaviors, acting as a social chameleon to adapt to different social situations, or using alcohol to feel more sociable.

Prior research has indicated that autistic people who experience identity-based stigma may camouflage their autistic characteristics or behaviors to assimilate to non-autistic cultural norms to achieve acceptance or success in different social spheres such as work, school, or relationships in addition to avoiding stigma (11). Despite potentially achieving this desired outcome (50, 51), autistic people have expressed that camouflaging can be highly stressful and anxiety provoking (11). Moreover, when people perceive that their autistic traits are flawed or faulty and need to be hidden, this can increase internalized stigma (49). Camouflaging in autism has additionally been linked to negative mental health and well-being outcomes including increased depression (36), suicidal thoughts and behaviors (52), increased stress and anxiety (53), and decreased sense of belongingness (54). A recent study by Perry and colleagues (10) found that while camouflaging did not mediate the relationship between autism stigma and decreased well-being, higher perceived stigma predicted greater reports of camouflaging which suggests that camouflaging is a response to stigma. An additional study by Bradley and colleagues (55) found that autistic people expressed utilizing camouflaging to cope with harmful societal labeling and a lack of acceptance and reported that extended periods of time spent camouflaging were exceedingly detrimental to their mental health despite the short-term positive impacts.

Camouflaging serves as a desirable option for autistic people to avoid differential treatment or prejudice, despite many reporting that they want to authentically present as themselves (47–49, 56). Based on the camouflaging literature, it appears that autistic people experience negative stereotypes and stigma regardless of their choice to either mask their autistic traits or disclose their autistic identity (47). These findings all suggest that many autistic people have rich self-perceptions and are keenly attuned to the consequences of appearing different than people across social contexts. The extant and growing research in both the camouflaging and stigma literature warrant the application of established theories that can help explain the integration of social identity, stigma and camouflaging, and stress and mental health outcomes.

### Theories of disability

Professionals have predominantly used two models to help conceptualize the experiences of disabled people: the medical model and the social model of disability. These models provide frameworks for how professionals, caregivers, and lay people understand and interact with disabled people across various settings. Despite having very different conceptualizations, both models are still implemented today with the goal of supporting disabled peoples’ quality of life.

#### Medical model of disability

Extant research has contributed to the understanding that the autism phenotype is exceedingly heterogenous; however, autism is often described using an etiological and deficit-based framework (57). Interventions often focus on only particular presentations of autism despite its heterogeneity, leading to difficulty with assessing intervention efficacy (58). This framework and conceptualization of autistic people is known as the medical model of disability (59). In the medical model, disability is defined as a pathological impairment within a person’s cognitive, social, or physical functioning (60). The goal of treatment focuses on the amelioration or cure of the within-person disability. Upon its first introduction, the benefits of the medical model included a decreased sense of shame and stigma related to disability, increased trust in medical or clinical professionals in supporting disabled people, and increased medical and technological advances (60). The medical model promoted care and services for disabled people, but it did not originally include disabled people in decision making on intervention or policy.

In the present day, the model is still very present in medical systems, such as the Diagnostic and Statistical Manual of Mental Disorders (1), as it helps professionals identify specific areas where disabled people may need support, and in facilitating clear billable areas for insurance purposes (61). Additionally, many etiological theories of autism—both biological and psychological—have arisen from the medical model.

While aspects of the medical model continue to be used in current research and practice, the model itself has been criticized for emphasizing within-person deficits, prescribing methods to assimilate autistic people into engaging in more “societally acceptable,” non-autistic norms, or in some extreme cases, aiming to cure or eliminate autistic traits altogether. Importantly, many autistic self-advocates have challenged the medical-focused conceptualization of autism. They maintain that, given autism’s socially and behaviorally based diagnosis and interventions, understandings of autistic people should similarly account for social, societal, and behavioral influences (62).

#### Social model of disability

The social model explains how autism can be both a within-person disability that affects a person’s daily functioning and a social identity that feels further disabling due to the limiting and biased beliefs of society (63). This model was developed by disabled people in the 1970s and 1980s in response to the civil rights and disability rights movements. Oliver (63) originally posited that disability is not solely a reflection of the deficits of an individual, but it results from a disabled person functioning within an unaccommodating environment or biased society. Furthermore, the social model asserts that everyday difficulties are not simply the fault of the individual but rather a broader failure of society to provide appropriate support or services to all people regardless of ability.

This environment paved a path for the emergence of the neurodiversity movement (64), a sociopolitical initiative ignited by autistic people communicating online and establishing a sense of community (3, 65). The movement’s central premise holds that differences in neurological functioning and development are a part of natural human variation, and there is no one normal or healthy type of brain or one right style of neurocognitive functioning (66, 67). The goals of the neurodiversity movement align with the disability rights movement as they both aim to eradicate stigma associated with neurological differences. Moreover, this movement aims to communicate that there has been a long history of both medical and social misunderstanding and maltreatment of neurodivergent people that has caused a great deal of suffering for them (68).

Autistic self-advocates have been reframing their understanding of their disability through the social model of disability (69–71). Through this reframed understanding of autism as an identity inspired by the neurodiversity movement, autistic people may view their disability as a marginalized or minoritized identity in a similar way that people think of their race, sexuality, or gender (72–74). By situating their autism as both a disability and an identity, this conceptualization can allow autistic people to establish their own feelings and beliefs about their diagnosis which consequently gives them a greater sense of autonomy and dignity (75). Despite the strong self-advocacy for implementing the social model of disability, the medical model of disability remains pervasive in research and clinical practice which can cause discordance between the autistic community and non-autistic family members or professionals (76). The social and societal implications for differently conceptualizing and discussing autistic traits and people can lead to in-group and out-group thoughts and behaviors that may have direct implications on autistic peoples’ self-perceptions and mental health.

### Identity-based theories

The increase of focus on autistic community, identity, and pride should be reflected in the way non-autistic researchers and professionals conceptualize autistic peoples’ experiences. The concept of social identity and its impacts is a complex, mechanistic relationship, and thus, this should be reflected in the way autistic identity and its correlates are studied. It is imperative to understand both how autistic people view themselves as well as how they are affected by non-autistic peoples’ treatment of them. Two identity-based theories—social identity theory and minority stress model—can bridge the current gaps in the autism literature on identity, stigma, and camouflaging by accounting for these complex interactions in one framework. Furthermore, these theories can inform how unique identity-related stressors, internalized self-perceptions, and maladaptive coping strategies may decrease mental health and well-being in autistic people.

#### Social identity theory

Social groups, norms, and their interactions have direct effects on the disparities for marginalized people within education, healthcare, employment, and community environments (77, 78). While a person’s social identity can give them a sense of belonging and understanding of themselves, it can also lead to the categorization of people within social dynamics (79). Social identity theory was first posited by Tajfel and Turner (80) to describe circumstances in which people see themselves as individuals or as members of a particular group. They additionally studied the consequences of a person’s personal or social identity and how this impacts self-perceptions and group behavior (13, 81). In the seminal studies, participants were assigned to arbitrary and meaningless groups and asked to assign points to other participants. Results indicated that participants systematically chose to award points more often to in-group members than out-group members. The researchers inferred that the simple act of categorizing people into groups can sufficiently lead people to see themselves as group members rather than as separate individuals. In turn, group membership can help people define their personal identity and decide how they relate to those around them.

Tajfel and colleagues’ initial studies asserted that group membership instills meaning in social situations, which inspired the development of social identity theory (13). This integrative theory combined cognitive processes and behavioral motivation, and initially focused on intergroup conflict and relations. Per the cognitive framework for social identity theory, the central psychological processes include social categorization, social comparison, and social identification. Social categorization refers to peoples’ propensity to place themselves and others into social categories. Social comparison is when people assign a relative value to a particular group or member. Lastly, social identification occurs when people view others through the lens of themselves and how they relate to others. The three processes result in social identity, or one’s knowledge of belonging to a specific group. Behavioral motivation is driven by both personal and group factors. Based on social identity theory, people attribute positive traits, attitudes, and behaviors as characteristic of their in-group members and less favorable qualities of the out-group. This difference in perception leads to disparities in outcomes, evaluation, performance assessment, and communication between the in- and out-group members.

Extant research has expanded upon the socially relevant outcomes resulting from social categorization including negative evaluations of out-group members (82), stereotyping (83), and lack of resource allocation to out-group members (84). Moreover, research has demonstrated that social identification can also be related to positive in-group bias (85). From both perspectives, the in-group treats the out-group poorly based on the motive to protect or enhance their own self-identity (13). While much of this early research focused on the in-group thoughts and behaviors, this treatment can infer a threat to the out-group members. Social identity threat is defined as the concern out-group members experience when the positive perception of their in-group is threatened by the presence of negative group stereotypes, devaluation of their members, or external stigmatization of their in-group (86). Prior research on social identity threat has demonstrated negative stereotypes towards women [e.g., (87)], older adults [e.g., (88)], immigrants [e.g., (89)], and people of low socioeconomic status [e.g., (90)], and these negative attributions can contribute to sustained inequality for marginalized groups in society. In addition to affecting performance [e.g., (91)], social identity threat can increase avoidance of or disengagement with a target domain [e.g., (92)] as well as be viewed as detrimental to the quality of one’s social life (93).

#### Minority stress model

While social identity theory explains in-group and out-group thoughts and behaviors, it is necessary to further understand the impact that systemic factors have on marginalized groups, like autistic people, who are historically oppressed in education, workplace, clinical, and personal settings. The minority stress model supplements the social identity theory by outlining and explaining the disparities that exist specifically between stigmatized groups and majority groups (14). Meyer (14) coined the term, “minority stress,” in response to conducting a literature review and meta-analysis on the prevalence of mental health concerns in lesbian, gay, and bisexual people and defined it as mental health problems that arise from prejudice, discrimination, or stigma that is present in hostile or stressful social environments. Additionally, the model asserts that stress processes exist both within an individual and as a function of the influences of broader social contexts. The model begins by situating minority stress within a person’s environmental circumstances which overlaps with a person’s minority status. Minority status has a direct relation to a person’s self-perception or self-identification. Environmental circumstances can lead to experiencing stressors including general stressors as well as stressors unique to minority group members such as distal events like discrimination in education, the workplace, or healthcare and proximal events like expectations of rejection or internalized bias. Lastly, a person’s minority identity can also moderate the impacts of stress in both positive and negative ways. Taken together, these processes all function to explain unique positive and negative mental health experiences for people with marginalized identities.

This framework functions on the basis that stigma and falling lower on the social hierarchy leads to a greater likelihood of experiencing greater stress or other mental health concerns while having less access to resources to cope with these occurrences (94). The additional presence of a tiered social structure facilitates discrimination and social exclusion which can add further stress to stigmatized groups. The model functions under four additional premises. The first principle states that differences between groups do not necessarily correspond to discrepancies when they are expected such as certain ailments more commonly occurring with older age. Second, social disadvantage does not need to affect the entire social subgroup, and if an individual person within that minority group does not experience it, it does not discredit the theory. The next premise is that the minority stress model applies broadly to social situations and overall health rather than to a particular disorder. Finally, the minority stress model specifically relates to sociological disadvantage influenced by external factors rather than representing a within-person difference or negative outcome.

The minority stress model has been historically implemented in the sexual and gender minority literature, which has demonstrated greater stress related to individuals’ identities and higher instances of poor physical and mental health (14, 95–98). Health disparities have also been linked to identity-related stressors in other marginalized groups including Black Americans (8, 99), undocumented Latinx immigrants (100), and physically disabled people (73). It is notable that up until 2020, no research had been conducted to apply the minority stress model to people with any type of neurodivergent identity.

### Applying identity-based theories to autistic people

Autistic people have qualitatively reported feeling different from others, lacking a sense of fitting in or belongingness, and feeling isolated and inferior to others [e.g., (101, 102)]; however, few studies have quantitatively assessed autistic identity and social categorization as catalysts for mental health or well-being (79, 103). Studies have demonstrated that autistic adolescents and young adults experience higher instances of depression resulting from feelings of loneliness (104, 105). Additionally, loneliness has been shown to be a strong predictor of depression in non-autistic populations [e.g., (106–108)]. While loneliness may appear conceptually similar to social identification, the two are separate constructs such that loneliness relates to a general disconnect between people whereas social identification relates more to feelings of belongingness to a particular group. Crompton and colleagues (109) conducted a qualitative assessment of autistic adults’ belongingness with each other and their well-being. They found that autistic people reported that spending time with other autistic people provided a sense of belonging as they were able to be their authentic selves and felt understood by other autistic people, which participants believed was important for maintaining their well-being.

Cooper and colleagues (103) were among the first to use social identity as a primary variable assessed within autistic peoples’ experiences. They asked autistic adults about their social identification with other autistic adults and its relation to self-esteem. Their path analysis results indicated that increased feelings of social identification with other autistic people predicted greater self-esteem towards their social group which in turn was predictive of greater personal self-esteem. When controlling for both forms of self-esteem as mediators, social identification was negatively associated with both anxiety and depression. Implications from this initial study indicate that feelings towards autistic people can influence both an autistic person’s self-perceptions and mental health outcomes.

Maitland and colleagues (79) expanded on this work by assessing how to measure social identification in autistic people, how autistic people relate to other social groups, and finally, whether social identification associates with depression, anxiety, and positive mental health. They found that measures of social identification originally developed for non-autistic populations showed good reliability but yielded a different factor structure when applied to autistic people, suggesting that they may experience social identity differently, but can still accurately report on their feelings towards it. Their findings showed that some autistic people identified with other social groups such as autistic people, their family, and other groups they had frequent contact with (i.e., work, peer, and hobby groups), and some autistic people felt as though they did not identify strongly with any group. It is important to note that this study did not have a diverse enough sample to assess what other social identity groups they identified with, such as gender, sexuality, race, or ethnicity. Socially identifying as autistic did appear to have a protective factor as it was reported to relate to lower levels of depression and higher positive mental health. Again, this study tended to focus on autistic peoples’ self-perceptions and belonging to their own group and less towards their feelings and perceptions of how non-autistic people perceive and treat them.

The current autism research informed by social identity theory has primarily focused on autistic group belonging and mental health. This does not account for the in-group and out-group dynamics and its subsequent impact on cognitive processes and behavioral motivation. Autistic people have been historically and systematically treated as an out-group, and thus, they likely experience being minoritized by society in social situations in similar manners to other marginalized groups. Therefore, the introduction and application of the minority stress model can supplement social identity theory research by framing autism as a minority identity that experiences specific stressors beyond being an out-group in social dynamics.

Several studies have demonstrated that autistic people are more likely than non-autistic people to have increased rates of physical and mental health concerns (34, 110–113), including greater rates of depression, post-traumatic stress disorder, and suicidality (111, 114, 115), which indicates a clear mental health disparity between the two social groups. Given this discrepancy, it is worthwhile extending the minority stress model to autistic people, since a tenet of applying the framework is that there must be documented disparities currently existing between the stigmatized and majority group (94).

Additionally, autistic peoples’ experiences can be applicable to the model structure that Meyer (14) initially proposed. First, autistic peoples’ neurominority status is intertwined with their experiences of identity-related stress in various social and environmental contexts (59). In line with the minority stress model, autistic people have expressed that holding a neurominority status relates to how they self-identify and see themselves (71). Moreover, autistic people experience unique minority stressors that extend beyond universal stressors such as prejudice from classmates in school settings (116), the workplace (56), and healthcare settings (117, 118). The final piece of the minority stress model in which social identity moderates stress has previously been absent within the autism literature.

Botha and Frost (17) conducted the first study to assess the impact of minority stress, above and beyond general stressors, and how it relates to autistic peoples’ mental health experiences. Their study comprised autistic adults from the United Kingdom who answered questions regarding stress, discrimination, camouflaging, stigma, and well-being. All models controlled for the influence of gender and general stress exposure. Results for the first model indicated that lower social well-being was significantly predicted by greater levels of both expectation of rejection and behavioral concealment. Next, lower levels of emotional well-being were significantly predicted by greater levels of victimization and discrimination, everyday discrimination, expectation of rejection, and internalized stigma. Lower psychological well-being was predicted by greater levels of victimization and discrimination, everyday discrimination, expectation of rejection, and outness. Finally, greater levels of psychological distress were significantly predicted by greater levels of everyday discrimination, expectation of rejection, outness, and internalized stigma as well as having an official autism diagnosis.

While these results were preliminary, they suggest that the minority stress model could be applicable to understanding autistic peoples’ mental health both theoretically and empirically (17). The findings support that autistic people experience unique stressors related to their identity that have an additive effect to other general stressors and make a strong argument that there is a need for this important research gap to be filled. Future directions of this research can more broadly explore and parse apart what the experiences of stigma, both external and internal, are like for autistic people, how their self-perception influences masking their autistic traits, how community connectedness or belonging could buffer mental health outcomes, and how other minority identities may have a “double discrimination” effect (119).

Autistic self-advocates and allies have been encouraging researchers to more broadly apply themes of acknowledging autism as an identity and minority status within research (15, 16); however, most of this research has been led by autistic researchers (17), who have reported that influential forces, like funding mechanisms and senior researchers, can make it feel emotionally taxing or professionally difficult to lead this research in a lower position of power (120, 121). Given that the use of the minority stress model to inform autism research is so nascent and led by members of the autistic community, non-autistic researchers in positions of power have the potential to positively impact and drive this research area forward to better understand minority stress in the same way that sexual and gender and racial/ethnic minority research has progressed. Moreover, conducting research that understands the impact of minority stress and how non-autistic people have intentionally or unintentionally perpetuated it dovetails well with a recent systematic review and meta-analysis on the interaction between non-autistic people’s characteristics and their attitudes towards autistic people (122). Their results indicated that gender, knowledge on autism, quality of contact with autistic people, and how many times they have interacted with autistic people can significantly predict how positively or negatively they view the autistic community. More frequently implementing the minority stress model in autism research prioritizes the initiatives of autistic self-advocates and researchers while directly increasing our understanding of both internal and external stressors and how they impact a person’s mental health and well-being.

The integration of social identity theory and the minority stress model interplay well together when investigating camouflaging, stigma, and mental health outcomes. Social identity theory asserts that groups use both individualistic and collective strategies to achieve a positive status (123). Camouflaging may serve as an individualistic strategy to separate from an autistic person’s in-group and be accepted into the majority-status and non-stigmatized non-autistic out-group. Additionally, Botha and Frost’s (17) findings demonstrate that autistic people have a stigmatized minority identity that is subject to specific stressors beyond everyday discrimination and prejudice. Therefore, a model integrating these two theories could examine the relationships between autism identity-related stigma and well-being with a mediating factor of camouflaging strategies while controlling for demographic factors and other general life stressors.

## Discussion

The present integrative theoretical review proposes a reframing for our understanding of autistic people and their disparities in mental health outcomes. The integration and application of social identity-based frameworks shifts the locus of difficulties and negative outcomes from being predominantly within an autistic person to being a mutual interpersonal issue between both the autistic and non-autistic person. This shared breakdown in understanding was defined by Milton (124) as the “double empathy problem.” This theory suggests that when people with different identities interact with each other, they may struggle to empathize with each other’s perspectives or experiences. Recent research has begun to account for the impact that non-autistic people contribute to social interactions [e.g., (23, 125–128)]; however, this has not yet been more widely applied to how researchers frame mental health outcomes for autistic people, how the double empathy problem plays a role in camouflaging, or how systems can be changed to reflect this knowledge.

### Implications for future research and practice

Suggestions for future research center around taking a more integrative and holistic approach to theories of autism. As previously mentioned, much of the current literature focuses on studying social identities, autistic traits, mental health, and camouflaging in separate studies. Across these studies, research has demonstrated that (1) social identity influences how autistic people view themselves and how others view them [e.g., (79)], (2) social exclusion can lead to poor mental health outcomes [e.g., (93)], (3) people with other marginalized identities have greater mental health concerns [e.g., (129)], (4) camouflaging to fit in can be physically and emotionally taxing [e.g., (11)], and (5) autistic people from other historically marginalized groups experience health disparities [e.g., (130–133)]. Future research is needed to integrate these areas; such research can account for these complex theories by utilizing more advanced statistical approaches like structural modeling and implementing person-centered approaches like mixed methods research. Additionally, non-autistic researchers should actively take an anti-ableist approach to their research by using more socialized frameworks of autism, including autistic people throughout their research process, and changing their language surrounding autistic people and their experiences (134).

This review also has important implications for intervention, education, and diagnostic practices, as well as broader implications for how professionals and lay people conceptualize and understand autistic peoples’ experiences. First, education-based programs and interventions should be more widely implemented to reduce the identity-based stigma perpetuated by non-autistic people (135). These programs can address implicit bias, microaggressions, or outward discrimination in multiple settings and can assist with developing more equitable and sustainable disability policy. Multiple studies have demonstrated that stigma reduction programs can increase autism knowledge and reduce autism stigma at the individual level in non-autistic adolescent and young adult samples (136–139). At the systems level, stigma reduction programs can help reframe the conceptual view of autistic people to reduce stigma and camouflaging in the workplace, school, and other public settings (140). One such intervention that has been proposed focuses on educating non-autistic people on the social model of disability through placing less emphasis on assimilating autistic people to non-autistic cultural norms and practicing greater acceptance (62). Bottema-Beutel and colleagues (140) also recommended adapting social skills interventions to shift focus from using normative, non-autistic social interaction norms as target outcomes to appraising realistic social skills goals to communicate in a way that best fits each individual’s needs and preferences.

Social identity and disability culture frameworks can also aid in addressing gaps in gold-standard diagnostic practices. The Autism Diagnostic Observation Scale-2 (ADOS-2) is a best-practice measure that focuses on behavioral observations to assign an autism diagnosis (141). Given that the measure can only be scored based on what is observed during the assessment, an ADOS-2 administrator cannot account for the presence and influence of camouflaging, and autistic people who engage in camouflaging may appear during the assessment as though they do not meet the diagnostic criteria for autism [e.g., (142, 143)]. Missing out on an autism diagnosis can lead to delaying access to supports or accommodations which can affect a person’s feelings of competence, belonging, and autonomy (144). Additionally, limitations of behavioral observational measures due to camouflaging contribute to disparities in diagnostics for people of color or sexual and gender minority people which has negative implications for their mental health (132, 145, 146). One way to supplement the ADOS-2’s observational approach is to include self-report measures of a person’s perceived autistic traits, such as the Autism Quotient (AQ) (147), or camouflaging, such as the Camouflaging Autistic Traits Questionnaire (CAT-Q) (54). While caregiver-report measures can also assist in a more holistic diagnostic assessment, parents or caregivers may be unaware if their child engages in camouflaging. A prior study found that non-autistic children as young as 5 years old can reliably and validly report their health-related quality of life (148), and psychometric research has identified an approach to estimate the minimum age that children can self-report data of similar quality to their parents or caregivers (149). This approach can be taken to assess and potentially adapt the AQ or CAT-Q to determine what age autistic children can validly and reliably self-report their autism traits or camouflaging. Moreover, better professional development and education on stigma and camouflaging can improve diagnosticians’ assessment and case conceptualization of clients.

This education can further benefit autistic people in therapeutic settings. Given the high co-occurrence of mental health concerns among autistic people, it is pivotal to change the stigma and barriers to systems of support. Brede and colleagues (150) found that the three most common themes among studies of mental health service experiences for autistic people included (1) a lonely, difficult service experience that can cause further harm, (2) a need for a more flexible and comprehensive approach to autistic mental health, and (3) listening to autistic clients, building strong and trusting rapport, and empowering their agency. In order to create safer and more trusting environments for autistic people to utilize mental health services, clinicians should actively work to dismantle their implicit biases that may unintentionally be harming autistic people and preventing them from seeking support. Additional exploration should focus on how to include autistic people with co-occurring intellectual disability or who are from historically marginalized racial, ethnic, or socioeconomic backgrounds to further identify even broader structural barriers to accessing equitable mental health care.

Chapman and Botha (151) emphasized the importance of clinicians adopting a neurodiversity-affirming therapeutic approach when working with autistic clients, as other classic theories of psychotherapy may not adequately capture an autistic clients’ experiences or support their goals. Neurodiversity-affirming therapy encourages therapists to (1) reconceptualize dysfunction as external rather than within-person, (2) emphasize the importance of autistic community, acceptance, and pride, and (3) adopt a cultural humility for disability and neurodivergence. It is also important for therapists to recognize the deleterious effects that camouflaging may have on their clients’ mental health (11), and to acknowledge the harmful impact of repeated experiences of stigma as a form of trauma when conceptualizing their clients’ therapeutic goals (152).

### Future directions

Future research and practice can integrate an identity-based theoretical framework with the autism and mental health literature to help providers and family members better understand and accommodate autistic people across their lifespan in multiple settings. As previously mentioned, very few studies to date (10, 17) have incorporated identity-based frameworks to understand autistic mental health, and the autism and stigma literature is less than 10 years old; therefore, this research area is relatively nascent in its development and dissemination.

#### Increasing inclusivity in stigma-related research

Despite the neurodiversity movement asserting the importance of all neurodivergent perspectives being included in research and advocacy, autistic people with co-occurring diagnoses remain underrepresented in the identity and stigma literature. The neurodiversity movement and the resultant sense of community has predominantly been facilitated through online communication (3). Additionally, many of the referenced studies included online surveys that required participants to read and complete self-report questionnaires and/or engage in interviews [e.g., (10, 17)]. The continued use of online platforms to conduct research allows for increased accessibility to participation for minimally speaking and non-speaking autistic people without co-occurring intellectual disability; however, many studies did not have participants self-report on their expressive language ability. To accurately capture the identities of autistic participants, studies should include additional demographic questions on expressive language and co-occurring diagnoses and increase the visibility and inclusion of autistic people with co-occurring conditions that may make it difficult to engage with online communities. It is also important to adapt or develop new assessments of perceived stigma and identity that can be completed by autistic people with co-occurring intellectual disability or cognitive or communication difficulties to capture their insights into their mental health experiences (153).

#### Incorporating mechanistic relationships

An additional limitation of much of the current literature is that it does not address potential mechanisms linking identity, camouflaging, and mental health outcomes. Future directions in this line of research should focus on taking a mechanistic approach to understanding the potential relationships between identity-based theories, camouflaging, and mental health outcomes. An initial area of exploration is the additional influences of other identities like race or gender. Autism research has traditionally comprised predominantly white, educated, higher socioeconomic status samples, and underrepresented autistic people with marginalized identities have often been excluded (154). Botha and Frost (17) acknowledged that they had too small of a sample size to further investigate the impacts of gender and race/ethnicity on experiences of camouflaging, identity-based stigma, and well-being. Additionally, follow-up research by Cooper and colleagues (155) assessed how autistic people relate to other social identity groups, namely gender. They found that autistic adults reported lower social identification with gender norms compared to non-autistic adults which is concordant with recent findings that autistic people are six times more likely to be transgender or gender diverse than non-autistic people (156). This may indicate that autistic people may more strongly identify with their identity than the social norms associated with it. Given these preliminary findings, future research must expand recruitment efforts to make autism research more accessible for and generalizable to autistic people of all backgrounds and lived experiences. A future direction of this research should include how demographic factors mechanistically play a role in affecting peoples’ mental health and subsequently how therapy can be sensitive to the interplay of these experiences.

Another mechanistic approach would be to explore how other stress models may explain how autistic identity and community can relate to stress and health outcomes. One idea would be to explore how expressive suppression and cognitive reappraisal relate to camouflaging. Cai and colleagues (157) were the first group to examine emotion regulation as a transdiagnostic factor in autistic people. They found that autistic people using low reappraisal and high suppression were more likely to have higher depressive symptoms and lower well-being. Additionally, they found that continuously using suppression strategies could be buffered by continued use of reappraisal. Emotion regulation strategies may help to predict which people are more likely to engage in camouflaging and when camouflaging would more likely lead to negative mental health outcomes. For example, a study from van der Linden and colleagues (158) found that autistic people had stronger emotional stress reactivity in a negative stress model affect than non-autistic people in response to daily life stressors. Given that identity-based stigma can be a daily life stressor, this model may explain how those stressors can translate to the negative mental health outcomes seen in camouflaging and stigma studies. An additional theory not explored in the autism literature is how social allostatic load, or chronic stress-induced diminished regulatory systems, may affect stress within interpersonal relationships (159). Overall, it is crucial to understand both what leads autistic people to experience identity-based stigma, camouflaging, and negative mental health outcomes, as well as what maintains it.

#### Reconstructing stigma

Stigma research in autism has focused on stigma as one large construct (37). While it is important to know that stigma broadly has negative implications on health and well-being, different types of stigma may have different effects on subsequent coping strategies or outcomes. Research from Pryor and Reeder (160) separated stigma into four primary types: self-stigma, public stigma, stigma by association, and structural stigma. Researchers have translated this framework to other populations including people with HIV/AIDS (161) and people with fetal alcohol spectrum disorders (162). Turnock and colleagues (37) mention internalizing stigma and the effects of stigma on the systems in which autistic people exist; therefore, a next step in autism research is to separate the types of stigma and mechanistically understand what types of stigma trigger camouflaging or lead to more negative mental health outcomes.

Moreover, it is critical to draw a distinction between stigma and discrimination. Stigma focuses on the internalization of biases which can place the onus of change on the marginalized person. Discrimination and prejudice are constructs that put greater emphasis on the harmful impacts of oppressive systems, beliefs, and practices on marginalized peoples’ well-being. In order to change the narrative surrounding autistic mental health and well-being, it is important that researchers do not conflate these separate experiences thereby drawing attention away from the detrimental impacts of external systems.

#### Modifying interventions and supports

Another way to support autistic people in improving their well-being is to improve upon current interventions and accommodations. It is critical to extend the extant research literature on stigma and mental health outcomes to identify ways to ameliorate negative experiences for autistic people. Strength-based interventions may provide an opportunity for clinicians to de-stigmatize identity-related deficits and focus on individual strengths (163). This can serve as a way for autistic people to collaborate on their therapy initiatives in a person-centered manner. Moreover, while strengths-based approaches have increased over the past several years, autistic and non-autistic researchers on an expert panel (163) identified that some current strengths-based approaches still stigmatize autistic people and have goals that are based on non-autistic social norms. Therefore, investigating how to destigmatize and improve the goals and structures of established interventions are important to the well-being of autistic people.

## Conclusion

The present integrative theoretical review explores how social identity theory and the minority stress model complement the frameworks of the social model of disability and neurodiversity movement. Additionally, integrating these theories allows researchers to better understand the high rates of mental health concerns in autistic people and that camouflaging can contribute to these issues. Constructing an identity-based theory of stress and mental health concerns for autistic people helps understand and address other diagnostic and clinical disparities for autistic people with multiple minoritized identities. This framework can be further applied to educational, clinical, and diagnostic settings and have broader implications for how non-autistic people think about autistic people.

## Author contributions

RR conceived the idea and drafted the first version of the paper. LB supervised the manuscript and contributed to the final version. All authors contributed to the article and approved the submitted version.

## Funding

RR received funding from the Organization for Autism Research through a graduate research grant (#2022G06). RR and LB were funded in part by the National Institutes of Health (#R21DC019715).

## Conflict of interest

The authors declare that the research was conducted in the absence of any commercial or financial relationships that could be construed as a potential conflict of interest.

## Publisher’s note

All claims expressed in this article are solely those of the authors and do not necessarily represent those of their affiliated organizations, or those of the publisher, the editors and the reviewers. Any product that may be evaluated in this article, or claim that may be made by its manufacturer, is not guaranteed or endorsed by the publisher.
